# Comparative Fatty Acid Profiling of Edible Fishes in Kuala Terengganu, Malaysia

**DOI:** 10.3390/foods10102456

**Published:** 2021-10-14

**Authors:** Annabella Tramice, Marco Trifuoggi, Mohammad Fadhli Ahmad, Su Shiung Lam, Carmine Iodice, Gennaro Velotto, Antonella Giarra, Sara Inglese, Adelaide Cupo, Giulia Guerriero, Giuseppina Tommonaro

**Affiliations:** 1 National Research Council-Institute of Biomolecular Chemistry, 80078 Pozzuoli, NA, Italy; ciodice@icb.cnr.it (C.I.); adelaide.cupo@gmail.com (A.C.); gtommonaro@icb.cnr.it (G.T.); 2 Department of Chemical Sciences, University of Naples Federico II, 80126 Naples, NA, Italy; marco.trifuoggi@unina.it (M.T.); antonella.giarra@unina.it (A.G.); 3 Faculty of Ocean Engineering Technology and Informatics, University Malaysia Terengganu, Kuala Nerus 21030, Terengganu, Malaysia; fadhli@umt.edu.my (M.F.A.); ge.velotto@studenti.unina.it (G.V.); 4 Higher Institution Centre of Excellence (HICoE), Institute of Tropical Aquaculture and Fisheries (AKUATROP), University Malaysia Terengganu, Kuala Nerus 21030, Terengganu, Malaysia; lam@umt.edu.my; 5 Department of Biology, University of Naples Federico II, 80126 Napoli, NA, Italy; inglese.eclab@gmail.com (S.I.); giulia.guerriero@unina.it (G.G.); 6 BAT Center—Interuniversity Center for Studies on Bioinspired Agro-Environmental Technology, University of Naples Federico II, 80055 Portici, NA, Italy

**Keywords:** Malaysian fish, fish identification, chemical biomarkers, fatty acid profiling

## Abstract

The aim of this study was to compare the relative nutritional benefit of edible Malaysian fishes from the coast of Terengganu in Malaysia, as well as to perform a taxonomical characterization and metal assessment. Discrimination between species was carried out by a morphological and molecular approach by evaluating the total concentrations of metals by ICP-MS analyses and the fatty acids (FA) composition using the GC–MS approach on the fish fillet tissues. The taxonomical studies detected fishes of 11 families and 13 species. The heavy metal assessment showed that all detected elements did not exceed the regulatory limit stated by Malaysian Food Regulations. The proportion of saturated fatty acids (SFA) ranged from 33 to 58.34%, followed by the polyunsaturated fatty acids (PUFA) values from 24 to 51.8%, and the lowest proportion was of monounsaturated fatty acids (MUFA), ranging from 12.7 to 35.9%. The ω-3/ω-6 PUFA and PUFA/SFA ratios were determined in the range 1.1 to 7.4 and 0.35 to 1.6, respectively. The C20:5 ω-3 and C22:6 ω-3 acids were detected at levels comparable to those found in the corresponding species from similar tropical marine ecosystems. The high FA values can be useful biochemical tools for comparing the relative nutritional benefits of these biodiverse and non-toxic edible Malaysian fishes.

## 1. Introduction

Malaysia is home to great marine biodiversity from the coastal area of the Malay peninsula extending to its islands and out to the sea. The wide range of fishes corresponding to different ecological niches favors the Malay fishing industry, regardless of whether it is for local food consumption or trade [1]. The growing popularity of consuming fish in Malaysia has been noticeable over the last decade [2]. Malaysian consumers have become more aware of the benefits of eating fish and consuming fish of high quality. Accurate species identification and toxicity assessment are fundamental when referring to edible fishes. The ability to correctly characterize organisms is rarely questioned when morphological and biochemical or molecular identification is reported [3,4,5]. Furthermore, the levels of lipids in fish can change in relation to the redox state [6] and metal-influenced lipid peroxidation [7,8]. It is known that heavy metals could influence the generation of reactive oxygen species (ROS) through different metal-mediated mechanisms, and free radical generation may affect the function of a great number of receptors and biomolecules [9,10]. An overexpression of ROS production induces oxidative damage, particularly lipid peroxidation, which can produce tissue damage and significant physicochemical alterations of cell membranes [8,11,12]. Oxidative stress interferes with the organoleptic and health quality of foods [13,14,15].

### 1.1. Fish as Source of Valuable Fatty Acids

Fishes are an important component of human nutrition because of the high quality of their lipid content, acting as a source of health-beneficial polyunsaturated fatty acids (PUFA), mainly eicosapentaenoic acid (EPA; C20:5 ω-3), docosahexaenoic acid (DHA; C22:6 ω-3), linoleic acid (LA; C18:2 ω-6), and arachidonic acid (ARA; C20:4 ω-6). The ω-3 PUFAs, mainly EPA and DHA, have been widely reported to produce several benefits for human health [16]. EPA and DHA are ubiquitous components of cell membranes, the developing brain, and the photoreceptors of fetuses and infants [17]. In aging populations, EPA and DHA have been shown to reduce the incidence of cardiovascular disease [18], heart failure [19], and sudden cardiac death [20], while providing protection against the deleterious effects of cognitive aging [17,21]. In addition, they improve brain and vision function and are involved in the prevention of cancer, arthritis, hypertension, and diabetes mellitus [22]. EPA and ARA are important for many metabolic and physiological functions and are precursor molecules for the production of eicosanoid hormones, which play a role in the inflammatory response, immune function, and regulation, as well as ionic regulation and reproduction in fish and mammals [23]. A deficiency in ω-6 PUFA linoleic acid leads to poor growth, fatty liver, skin lesions, and reproductive failure [24,25]. However, in ecological studies, fatty acids are considered very important biomolecules because they have been used as trophic biomarkers in the marine food chain analyses of several ecological niches [26]. This is related to the fact that specific fatty acids, in particular some polyunsaturated fatty acids, can be traced as essential dietary components to higher trophic levels, such as zooplankton [27] and fish [28], as they are biosynthesized only by their specific prey (phytoplankton and macroalga). In this sense, the methods for determining fatty acid profiles act as a complement to the use of stable isotopes of carbon and nitrogen [29] and DNA-based techniques [30] to investigate habitat ecology.

### 1.2. The Importance of the Taxonomic and Nutritional Characterization of Malaysian Fishes

Although several sources of information regarding the taxonomy and the distribution in different geographic areas of fish species in Malaysian waters are available [31,32,33], few studies have investigated the correlation between fish nutritional values and their taxonomic profiles [34]. In the present study, the fatty acid (FA) tissue compositions of 13 species of fish from the Malaysia Sea were determined. This is the first extensive lipid profiling involving many tropical edible fishes. Since these species are used in the diets of the local populations, it was reasonable to select the fillet muscle as the target of our investigation.

Our goal was to study the extent of the interspecific variation in the composition of FA between the selected edible species after their taxonomic characterization and metal assessment. These results could provide some indications on how to address the fishing needs of consumers in emerging countries, respecting the sustainable development of fishing and following the guidelines established by the United Nations FAO in the 2030 Agenda for the sustainable development of natural resources [35].

## 2. Materials and Methods

### 2.1. Site of Fish Collection

**Figure 1:**
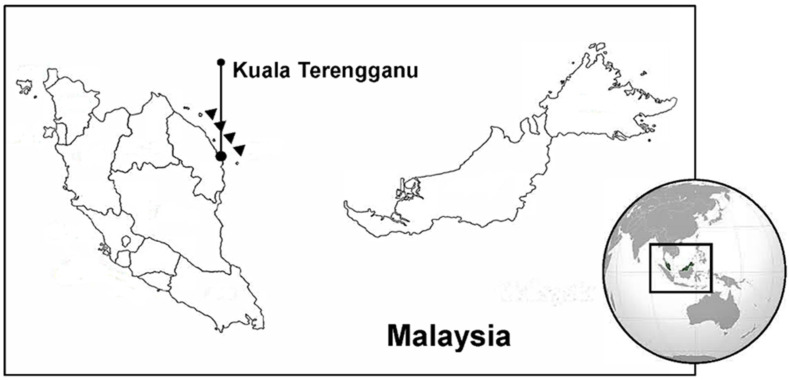
Location of the study site at Kuala Terengganu in the Malaysian South China Sea. Triangles correspond to distances from 12 nautical miles up to 30 miles off the coast.

Fishes were collected at Kuala Terengganu (Malaysia, Figure 1) in April 2019. Kuala Terengganu belongs to the State of Terengganu, which is located on the east coast of the Malaysian Peninsula. Terengganu borders Kelantan to the north and Pahang to the south, and the coast of Terengganu faces the South China Sea. It is made up of three named zones, the Western Belt, Central Belt, and Eastern Belt; and Kuala Terengganu is located in the Eastern Belt (Figure 1). Igneous rocks in the Eastern Belt were found to be mostly biotite, granite, and minor rocks of intermediate composition [36].

The different fish species were recovered by professional fishermen with bottom trawls from a distance of 12 nautical miles up to 30 miles off the coast. Twenty specimens of each fish species were examined, regardless of the number of samples received. After creating photographic records and conducting external morphological examinations, fish fillet tissues from the center of each body (Supplementary Figure S1) were dissected from each specimen and stored at −20 °C until molecular processing.

### 2.2. Identification of Malaysian Fish Species

The fishes were identified from the family to the genus level on the basis of these macromorphological characteristics: features of the head, body shape and color, eye diameter and position, opercle, lateral line, number, position and form of the fins, size and shape of the scales, shape and position of the mouth, and type of teeth [37]. The most updated keys for identification were used, specifically WoRMS ID [38], FishBase [39], and the FAO [37]. Fish images were then created using the Inkscape program [40] to show the anatomical characteristics detected for taxonomical identification. Molecular recognition was also conducted for all specimens on 100 mg of fillet using the barcoding method reported in Di Finizio et al. [4] using the following primers: COI_UP (5′- ACTTCAGGGTGACCGAAGAATCAGAA-3′) and COI_DW (5′-ATCTTTGGTGCATGAGCAGGAATAGT-3′) [41,42,43]. Using FASTA, the fragments of COI tRNA sequences obtained after amplification, purification, and sequencing were then compared to GenBank similar COI rRNA sequence data [44,45]. 

### 2.3. Metals Quantification

Metal analysis was carried out on a pool of fillet tissue of each species of Malaysian fish after acid digestion. Samples of 500 ± 5 mg were digested with 2 mL of ultrapure nitric acid (HNO_3_ ≥ 69%, *v*/*v*) in test tubes. The latter were placed in a water bath, preheated to 80 °C, and proceeded to digestion for 3 h. Samples were brought to a final volume of 10 mL with a solution of HNO_3_, 2%, *v*/*v* for the subsequent elemental analysis by inductively coupled plasma–mass spectrometry (ICP–MS, Aurora M90, Bruker, Billerica, MA, USA). The digestion step was performed using a blank sample to identify potential metal contamination of any of the materials and reagents used. A calibration curve was obtained for each analyzed element from a certified standard solution (Ultrascientific, Bologna, Italy). The limit of detection (LOD) was calculated by the method of blank variability for each investigated metal, and they were included in the range of 0.01–0.2 µg/g in the final sample. The accuracy was evaluated using Certificated Reference Material (CRM) of a fish-based sample from an interlaboratory comparison (QMAS, MT-281 Sample 742, LGC Standards Srl, Milano, Italy), obtaining a recovery in the range of 85–115% [46].

### 2.4. Lipid Extraction

Lipids were extracted by following the modified Bligh and Dyer [47] method. Each frozen fish sample was analyzed in triplicate. Fish tissue fillets (~4–13 g of samples) were immersed in 50–100 mL of chloroform: methanol (1:1 *v*/*v*), and lipid release was promoted by crumbling the tissues in a mortar for 5–10 min. The procedure was repeated three times with fresh aliquots of organic solvent mixture. The collected solvent fractions were filtered on filter paper, transferred in a separating funnel, and combined with distilled water (ratio 1:1 *v*/*v*). The organic layer was removed, dried over anhydrous sodium sulfate, and reduced in vacuo. The extracted lipids were also placed in a desiccator to remove residual solvent traces and weighed to determine the total lipid content in the fillet tissue of each sample.

### 2.5. Fatty Acids Analysis

The qualitative and quantitative characterizations of the total fatty acids recovered from the fillet tissue organic extracts were determined by GC–MS on the corresponding fatty acid methyl esters (FAMEs) obtained after the saponification of lipid extracts and following a modified AOAC Official Method, 991.39 [34]. The lipid fraction was derivatized to fatty acid methyl esters (FAMEs) by methanolic sodium methoxide anhydrous (2–4 mL, 1 N) at 90 °C for 55 min [48]. All operations involving lipids or their constituent fatty acids were conducted in an atmosphere of pre-purified nitrogen in order to minimize the oxidation of the polyunsaturated chains. FAMEs were recovered by adding diethyl ether (extraction with three aliquots of 1–2 mL each) to the cooled reaction mixture. The FAME mixtures were dissolved in diethyl ether (EE, 1 mgmL^−1^) and analyzed by GC–MS equipped with an ion-trap (Thermo Scientific, Waltham, MA, USA) on a 5% diphenyl-polysiloxane column (OV-5 column, VF-5ms 30 × 0.25, Agilent Technologies, Middelburg, The Netherlands), Thermo Scientific™ PolarisQ ™ GC/MSn Benchtop Ion Trap Mass Spectrometer in EI (70 eV), and positive mode analysis (mass range 50–450). The elution of free fatty acid methyl esters required an increasing temperature gradient according to the following method: 160 °C for 3 min, then 3 °C/min until reaching 260 °C and 30 °C/min until reaching 310 °C; the system was held at 310 °C for 5 min. Samples of 2 μL were directly injected in split mode (1:10) and a split flow of 10 mL min^−1^, with a blink window of 3 min (with an inlet temperature of 270 °C, the transfer line set at 280 °C, and an ion source temperature of 250 °C). The carrier gas was helium, which was used at a constant flow of 1.0 mL min^−1^. FAMEs were identified by comparison of their retention time with those present in a mixture of 11 standard FAMEs (marine source, analytical standard, Sigma Aldrich) which was analyzed in the same conditions. To monitor the performance of the column in the GC, the standard FAMEs mixture was chromatographed at regular intervals after every ten samples. Aiming to quantitatively characterize each fish fatty acid mixture, for each GC–MS measurement, an internal standard was added to a FAME solution to be analyzed; 50 μL of a 2.5 mg mL^−1^ solution of methyl tricosanoate (C23:0) was used, corresponding to 0.125 mg of internal standard per mL of the solution to be analyzed (0.238 μg for each injection of 2 μL).

The equation

CFA = [(m_IS_ × A_FA_ × RRF_FA_)/(1.04 × m_fis_ × A_IS_)]/V_-injection_

was used to quantify all measured fatty acids, where CFA is the concentration of the fatty acid in mgmL^−1^, V_-injection_ is the volume of each injected sample solution (2 μL), m_IS_ is the weight of the internal standard, A_FA_ is the fatty acid peak area in the GC spectrum, RRF_FA_ is the relative retention factor for each fatty acid [34], 1.04 is the correlation factor between the fatty acids and the fatty acid methyl esters, and A_IS_ is the internal standard peak area in the GC spectrum. The RRF value accounts for the effective carbon number, and it was calculated according to previously published methods [49]. The response factors for each of the FAMEs not present in the standard mixture were estimated by comparison with the standard FAMEs that resembled them most closely in terms of the chain length and the number of double bonds. Starting from CFA, it was possible to establish the total amount of each fatty acid (FA) contained in the recovered EE extract of each fish species; according to the weight of the frozen fillet fish tissue (m_fis_), the milligrams of each fatty acid in 100 g of fish fillet tissue were determined using the following formula:

[(mg FA in 1 mg of EE extract × total mg of EE extract)/g m_fis_] × 100

where g m_fis_ corresponds to the grams of fish fillet tissue analyzed. Three replicates of each sample were obtained. A total of 11 fatty acids were measured, with a limit of quantification (LOQ) of 0.2 mg/100 g of fillet.

### 2.6. Statistical Analysis

The results obtained were presented as the means of the measurements and standard deviation (SD) or standard error (SE). Significant differences in the contents of metals and fatty acids in the fillet of the studied fish were estimated using a one-way analysis of variance (ANOVA). Differences were reported as statistically significant at *p* < 0.05.

## 3. Results

### 3.1. Fish Identification

#### 3.1.1. Morphological Identification

**Figure 2:**
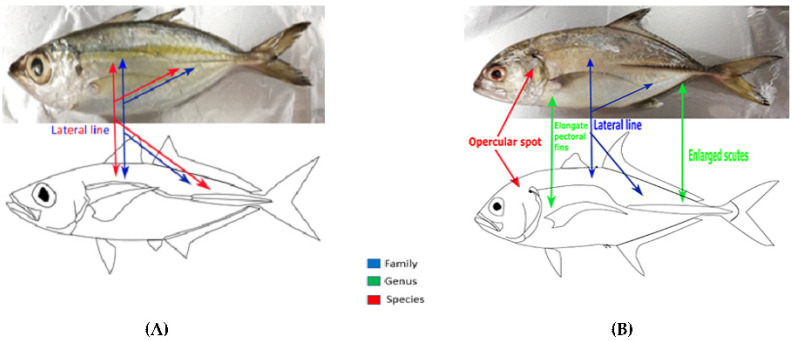
Photographs and drawings of fishes of the Carangidae family: *Selar crumenophthalmus* (**A**) and *Caranx sexfasciatus* (**B**). The colors of the arrows are indicative of the anatomical features.

The application of taxonomical criteria adopted to discriminate the fishes from family to species is shown in Figure 2.

Fishes with lateral lines elevated anteriorly and straight posteriorly, extending onto the caudal fin, were categorized as members of the Carangidae family. Fishes of this family were *Selar crumenophthalmus* and *Caranx sexfasciatus*.

Specimens in which we detected a shoulder girdle (cleithrum) margin with a furrow ventrally, a large papilla immediately above it, and a smaller papilla near the upper edge were identified as belonging to the genus *Selar*. The characteristics of a curved part of the lateral line with 48 to 56 scales, a moderately curved part of the lateral line with the chord of the curved part contained 0.7 to 1.2 times within the straight part; and smaller scutes showed that these fish of the genus *Selar* belonged to the species *crumenophthalmus*.

Specimens in which we detected characteristics of a posterior straight part of the lateral line with enlarged hardened scutes and long, falcate pectoral fins that were longer than the head were classified as belonging to the genus *Caranx*.

*Caranx* with dorsal fin lobes with white tips; a dorsal profile with the head moderately convex and a black spot on the upper margin of the opercle, a shorter postorbital head length contained 6.4 to 8.2 times in the fork length, and a longer dorsal fin lobe contained 5 to 6.6 times in fork length; and 10 + 15 vertebrae were classified as *sexfasciatus*. A similar analysis was performed for all fishes utilized in our studies. The tools of this morphological research consisted of determination keys, images, field guides, and other textual information. The photographs and drawings created for all species identified and their schematic anatomical characteristics are extensively described in Table S1 of the Supplementary Materials.

#### 3.1.2. Barcoding Sequences

The described set of different primers successfully amplified the corresponding mitochondrial region fragment of cytochrome oxidase 1 (COI) examined (data not shown) in all fish samples. Their PCR products isolated from the gel were sequenced. All sequences subjected to FASTA searches against the NCBI DNA database were classified according to the sequences with which they aligned with the highest identity, confirming the morphological identification of all specimens, representing 13 species and 11 families (Table 1).

### 3.2. Metals Accumulation

The concentration of 16 elements (Al, As, Be, Cd, Co, Cr, Cu, Hg, Li, Mn, Ni, Pb, Sb, Se, V, and Zn) was evaluated by ICP–MS analyses to assess any interspecific differences in the fillet tissue bioaccumulation.

Concentrations of non-essential elements such as antimony (Sb), beryllium (Be), cadmium (Cd), mercury (Hg), and lead (Pb) are shown in Table 2.

Among the analyzed species, the amount of Sb, Be, and Cd was below the limit of detection (LOD) for all the fishes.

Table 3 shows the concentrations of the group of essential metals consisting of copper (Cu), manganese (Mn), nickel (Ni), selenium (Se), and zinc (Zn), along with additional trace elements, which were elements without well-defined roles and may be essential [50].

Among the 12 species of analyzed fillets, the amount of Li and Ni below the LOD was detected only in *Scolopsis monogramma* (0.28 mg/kg).

**Figure 3:**
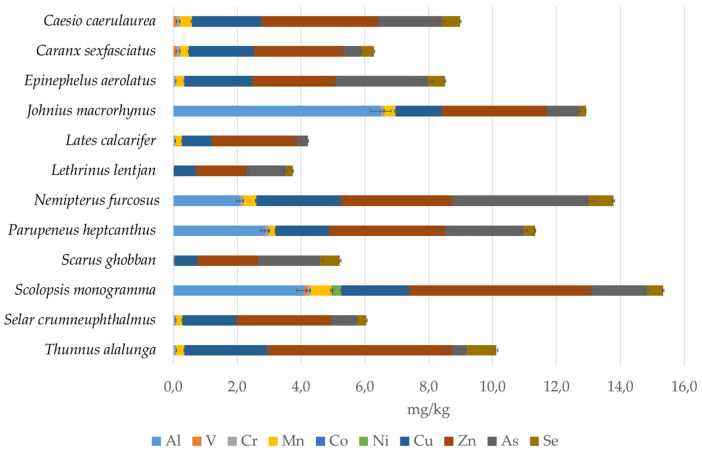
Interspecific differences in the contents of essential metals and additional trace elements in the fillets of different fish species (mean mg/kg of fillet ± SD).

Figure 3 reports the differences between the 12 species for the contents of essential metals and additional trace elements.

### 3.3. Fatty Acids Profile

The total lipid profile was determined for fillet tissues of all species collected in this study. A summary of the total fat, FAs, saturated fatty acids (SFA), monounsaturated fatty acids (MUFAs), and PUFAs (polyunsaturated fatty acids), with attention to ω-3 and ω-6 FAs, recovered for each species is presented in Table 4. Among the 13 species of analyzed fillets, the amount of total fat ranged from 1.84% (*Selar crumenophthalmus*) to 0.57% (*Parupeneus heptacanthus*); *Lates calcarifer* (1.56%), *Johnius macrorhynus* (1.16%), *Thunnus alalunga* (1.14%), *Lethrinus lentjan* (1.14%), and *Himantura walga* (1.12%) had the next highest fat content, and *Scolopsis monogramma* (0.64%) and *Caesio caerulaurea* (0.67%) had the other lowest values of total fat content.

Furthermore, the fatty acid profiles of the investigated fish tissues were also considered. The SFAs were the predominant fatty acid class and ranged from 14.2 ± 1.7 mg/100 g of fillet for *Epinephelus areolatus* to 360.2 ± 23.8 mg/100 g of fillet for *Himantura walga*. An evaluation of the percentage of SFAs of the total FAs furnished a range of values from 33 to 58.4%. The second highest class was the PUFAs, ranging from 10.5 ± 2.0 mg/100 g in the fillet of *Epinephelus areolatus* to 209.6 ± 13.2 mg/100 g of fillet for *Lates calcarifer*, corresponding to a range from 24 to 51.8% of the total FAs.

The proportion of MUFAs was the lowest, ranging from 4.5 ± 0.1 mg/100 g of fillet for *Epinephelus areolatus* to 241.4 ± 23.6 mg/100 of fillet for *Lates calcarifer,* corresponding to a range of 12.7 to 35.9% of the total FAs.

*Lates calcarifer* was found to be the species with the highest content of fatty acids, corresponding to 672.8 ± 46.3 mg/100 g of fillet. However, of this amount, 33% was comprised of SFAs (221.8 ± 9.5 mg/100 g), and only 31.2% was represented by ω-3 and ω-6 fatty acids (209.6 ± 13.2 mg/100 g). Interestingly, this was the highest content of ω-3 and ω-6 fatty acids recovered among the investigated fillet species. *Himantura walga* fillets contained 636.5 ± 47.7 mg of FAs per 100 g of tissue, of which 56.6% was comprised of SFAs (360.2 ± 23.8 mg /100 g), and 26.8% was represented by ω-3 and ω-6 fatty acids (170.7 ± 14.6 mg/100 g).

The species with the third-highest content of FAs was *Johnius macrorhynus*, with 474.6 ± 25.7 mg/100 g of fish tissue; 53.2% (252.2 ± 13.4 mg/100 g) were SFAs, and 12.40% consisted of ω-3 and ω-6 fatty acids (88.0 ± 10.2 mg/100 g).

*Himantura walga* was the species with the highest content of ω-3 fatty acids (127.8 ± 4.4 mg/100 g) among the investigated fish fillets, followed by *Lates calcarifer*, with 112.0 ± 4.3 mg/100 g of fillet, and *Thunnus alalunga*, with 95.4 ± 4.5 mg/100 g of fillet. *Thunnus alalunga* was also the species with the lowest amount of saturated fatty acids (SFAs) of the three species mentioned above, with only 85.1 ± 2.1 mg/100 g of fillet. However, the ω-3 fatty acids values were also interesting for *Selar crumenophthalmus* (76.3 ± 3.5 mg/100 g), *Scolopsis monogramma* (70.5 ± 5.3 mg/100 g), *Lethrinus lentjan* (60.6 ± 4.0 mg/100 g), and *Johnius macrorhynus* (58.9 ± 6.1 mg/100 g). In addition, *Lates calcarifer* and *Selar crumenophthalmus* fillets contained not only a good percentage of ω-3 fatty acids (16.5 and 19.1% of the total FAs, respectively), but these fishes were considered fatty fishes, with SFA contents of 221.8 ± 9.5 and 226.2 ± 5.2 mg/100 g of fillet, corresponding to 33 and 56.5% of the total FAs, respectively.

In Table 4, the ω-3/ω-6 PUFA and PUFA/SFA ratios are also reported: the ω-3/ω-6 PUFA ratio ranged from 7.4 ± 0.4 for *Nemipterus furcosus* to 1.2 ± 0.2 for *Lates calcarifer*, and the PUFA/SFA ratio ranged from to 0.35 ± 0.05 in *Johnius macrorhynus* to 1.6 ± 0.2 in *Scolopsis monogramma*.

## 4. Discussion

### 4.1. Fish Identification

Ocean products form the basis of many activities, such as the fishing industry, and are important for nutrition and well-being. The consumption of endemic fish has increased significantly over the last few decades, and specifically, the demand has also increased for fish, which is considered the most common source of lipids, proteins, and antioxidants. Today, the correct identification of fish species and their precise, updated, and taxonomic ordering, as well as the detection of metals (essential and non-essential) and their nutritional value, particularly in terms of lipid content, are the basis of most of the international fish trade [51]. In countries where the fish market is increasingly evolving, as well as in Malaysia, there are legal regulations that cover these points [52].

In addition, tools are needed to enable the rapid, reliable, and cost-effective identification of highly processed fishery products in the markets. In this study, 13 different fish species from Kuala Terengganu were preliminarily and morphologically identified with the aim of properly assigning the genus and species for their taxonomic characterization, as reported in Table S1 of the Supplementary Materials, and then genetically investigated. A dataset of 13 different COI sequences was obtained (Table 1) and compared with those already present in the GenBank data. Table 1 indicates that the barcoded specimens that were reliably assigned binomial names a priori possessed distinct COI sequences and could be aligned and referred to the sequenced specimens already published. As known, barcoding is defined as the use of a standardized short region of DNA to verify species identity, which for fish is typically the COI region of mitochondrial DNA, with the generation of publicly accessible and highly comparable data using GenBank [5,42,43].

Our data confirmed the taxonomy of widely distributed species present in Malaysia [53,54] and permits the identification of species of great interest for human consumption. Furthermore, these data highlight the importance of Kuala Terengganu for its richness in biodiversity. However, barcoding requires sophisticated laboratory instrumentation; it has a only small role in whole fish identification and limited use in other areas if not tied to taxonomic baseline data. In this sense, the availability of tools to identify images or morphological keys for non-experts (Table S1) would facilitate the classification and the possible direct marketing of products without adding further non-productive costs needed for barcoding [55].

Moreover, further molecular characterization of the collected fish fillets was carried out, and their metal concentrations and fatty acids (FA) profiles were determined. In recent years, studies on heavy metal bioaccumulation have been in progress, both in fish, marine waters, and seaweed.

### 4.2. Metal Accumulation

The results for the heavy metal content in the fish species in this study showed a low risk related to the accumulation of toxic elements, as has already been demonstrated in other aquatic organisms in Malaysia [56], in particular in Kuala Terengganu [57,58].

As food consumption is one of the main routes of exposure to metals [59], regulatory authorities in many countries have set limits for heavy metal concentrations in foodstuffs.

Our results indicate that the risk of exposure to heavy metals from the consumption of the Malaysian fish collected is relatively low and in compliance with the Malaysian Food Act 1983 and Regulation 1985 and with EU regulations.

In fact, five species (Table 2) showed the presence of Hg above the LOD, with a range of concentrations between 0.015 to 0.090 mg/kg, and nine species showed the presence of Pb in the range 0.021 to 0.10 mg/kg.

Concerning Pb, Cd, Sb, and Hg concentrations, the preliminary results presented here support the possibility of using these fish species for human consumption without hazard because the measured levels of these elements were found to be below the fixed limit of 1 mg/kg of wet weight. All samples were also below the maximum tolerable limit (MTL) defined by EU Commission Regulation No. 1881/2006 (Reg. 1881/2006), in which the limit values for fish meat, particularly for fillet, are 0.30 mg/kg for Pb, 0.050 mg/kg for Cd, and 0.50 mg/kg for Hg.

Among the detected metals, we also studied those which are essential elements (Table 3, Figure 3) according to the World Health Organization (WHO) classification, which considers their daily intake and nutritional importance [60]. The essentiality of an element is derived from its role in biochemical functions. Hence, if their daily intake is less than 100 mg/day, the resulting deficiency could cause a reduction in physiologically important functions [60]. All the species showed a high concentration of essential elements (Cu, Zn, and Se, up to 5.8 mg/kg), except for Mn, which was present in all species at a concentration between 0.2 to 0.68 mg/kg. The safe limits for concentrations set by the Malaysian Food Act 1983 and Regulation 1985 are 10 mg/kg for Cu and 100 mg/kg for Zn. There are no regulated limits for the other metals, except for inorganic As, which is fixed at 1 mg/kg.

In our results, As was expressed as total arsenic, so we could not compare our concentrations obtained in the samples to the allowed limit. The high toxicity of As is primarily due to the inorganic form, while organic forms of arsenic may be contained in fish and seafood, but these are less hazardous [61,62]. Metals are elements with significant roles as precursors in many enzymatic reactions. They have considerable importance in biological functions, and their deficiency can cause metabolic disorders [63,64]. Investigated metals have a redox potential contribution to the formation of ROS and subsequent lipid peroxidation [65], so we expect that a low metal content does not affect the lipid pattern in the investigated species.

### 4.3. Fatty Acid Profile

An extensive FA profiling was determined for fillet tissues of all species collected in this study. A summary of the total fat, FAs, SFAs, MUFAs, and PUFAs, with attention to ω-3, and ω-6 FAs, recovered for each species is presented in Table 4.

In general, the fats and fatty acid profiles in fishes vary with fish species; there are several factors responsible for this variation, such as the geographical location, season, food availability, water temperature, age, size of the fish, and maturation status [54,64,66].

It was previously discovered that levels of fatty acids also differ between latitudes, with species from high latitudes having higher amounts of PUFAs than tropical, low-latitude species [67,68]. In agreement with previous studies [26,33,54,69,70], the saturated fatty acids (SFAs) were the predominant fatty acid class and ranged from 32.96 to 58.38% of the total free fatty acids. In general, fishes recovered from warm seas tend to show high levels of SFAs with higher levels of palmitic and stearic acids compared to those from cold waters. This difference is due to metabolic differences between cold and warm water species because these fatty acids are not usually subject to differences in diet [67]. The lipid content percentage of *Lates calcarifer* (1.56%) was in agreement with findings in studies previously performed in species from the Vietnamese seas (1.7%) and showed clear differences with fishes recovered from different geographic areas, such as the Australian coasts (3.2%). Furthermore, the fatty acid (FA) profiles of fishes from different geographic areas were similar to our findings. For example, the Vietnamese species had levels of 24.8% for MUFAs, 41.0% for SFAs, and 13.3% for ω-3 FAs; in particular, this last value is very comparable to the 16.65% ω-3 FA level recorded in our fillets but very different from the 4.1% ω-3 FA value recovered in Australian species [71]. Concerning *Thunnus alalunga*, the total lipid content and free fatty acid composition (36.38% for SFAs, 17.20% for MUFAs, and 40.82% for PUFAs) is comparable with that of fish fillets from the Mauritius coasts in the western Indian Ocean [33]. In agreement with the abovementioned fatty acid composition of fishes collected in warmer waters, *Thunnus alalunga* specimens harvested off the U.S. West Coast have been reported to be less rich in SFAs than Malaysian ones, with a total ω-3 FA content averaging 40% of total fatty acids, which is very similar to our results [72]. The *Selar crumenophthalmus* fatty acids profile results were comparable to those reported by Metillo et al. [26] for species recovered in the Philippine seas; it should be noted that for the Malaysian species, an increase in the quantity of ω-3 and ω-6 FAs was recorded: 23.99% of total FA (96.03 ± 6.18 mg/100 g) compared with the previously reported 14.44% value [26].

Fish are able to synthesize SFAs and MUFAs de novo and selectively adsorb and metabolize dietary fatty acids, including PUFAs. The PUFA conversion capacity varies among fish species [73]. The ω-3/ω-6 PUFAs and PUFA/SFA ratios have been suggested as useful indicators for comparing the relative nutritional values of fishes [74,75]. PUFA/SFA ratios above 0.45 and ω-3/ω-6 ratios above 0.25 have been advisable by the UK Department of Health [76]. The best PUFA/SFA and ω-3/ω-6 PUFA ratios were recorded for *Thunnus alalunga*, *Nemipterus furcosus*, *Lates calcarifer*, and *Scolopsis monogramma*, as shown in Table 4. Our results should give some indication about the species which may be introduced into the Malaysian diet; in fact, considering the percentage of FA ω-3, *Thunnus alalunga* could be replaced by *Scolopsis monogramma* and *Lethrinus lentjan* because their fillets have an interesting amount of the ω-3 fatty acids (70.47 ± 5.29 and 60.61 ± 4.0 mg 3/100 g, respectively) and a relatively low amount of SFA (63.88 ± 2.97 and 197.50 ± 11.54 mg/100 g, respectively). An increase in the human dietary ω-3/ω-6 fatty acid ratio is essential to help prevent coronary heart disease by reducing plasma lipids and to reduce cancer risk; it also seems to be effective in preventing toxic shock syndrome and cardiomyopathy [77]. According to these benefits, the European Food Safety Authority (EFSA) recommends that adults (especially pregnant and nursing women and seniors) consume 227–340 g of seafood each week, which may provide 250 mg of EPA plus DHA per day [78]. These results could provide some indication about how to address the fishing needs of consumers in emerging countries, respecting the sustainable development of fishing and in accordance with the guidelines established by the United Nations FAO in the 2030 Agenda for the sustainable development of natural resources [35].

## 5. Conclusions

Fish is an important dietary component in many countries, and in 2017, fish consumption accounted for 17% of the world’s consumption of animal protein. The actual transfer and application of fish identification and nutritional evaluation technologies for the enhancement of Malaysian local fish products in management projects and schemes are overdue. An important objective of this paper was to promote the informed use of the Malaysian fish species of Kuala Terengganu, particularly regarding the nutritional lipid profile and possible metal contamination. In this sense, 13 fish species belonging to 11 families were taxonomically identified, and drawings were created for all species with the schematics of the anatomical characteristics described. Their fillet tissues were chemically investigated and characterized for the total metal content and lipid profile with particular attention to their FA composition. In general, all analyzed species showed a high concentration of essential metals and a low amount of heavy and non-essential metals, which are defined on the basis of their toxicity and the absence of a biological role. In addition, interesting values of PUFA/SFA and ω-3/ω-6 PUFA ratios were recorded for *Thunnus alalunga*, *Nemipterus furcosus*, *Lates calcarifer*, and *Scolopsis monogramma*. Overall, the results reported in the present study underline the need for different approaches when making evaluations about the nutritional quality of foods. In particular, an integrated study on the total lipid content and fatty acid composition, together with an evaluation of heavy metal content, is essential for providing nutritional information to the consumer and contributing to market foods in the category of healthiness and well-being.

## Figures and Tables

**Table 1:** Common names, scientific names, taxonomy, and relative GenBank accession numbers of the COI barcode sequences for Malaysian fish species discrimination in this study.

	English Common Name/Local Fish Common Name	Identified Species Taxonomy (*Genus, Species*, Family)	COI Genbank Accession Number
1	Blue and gold fusilier/ Delah biru emas	*Caesio caerulaurea* Caesionidae (Lutjanoidea)	JF492993.1
2	Bigeye trevally/ Belukok putih	*Caranx sexfasciatus* Carangidae	KY371313.1
3	Areolate grouper/ Kerapu bintik bulat	*Epinephelus areolatus* Serranidae	KJ202152.1
4	Dwarf whipray/ Pari ketuka tanjung	*Himantura walga* Dasyatidae	KF604912.1
5	Big-snout croaker/ Gelam bangkok	*Johnius macrorhynus* Sciaenidae	KX777976.1
6	Barramundi/ Siakap putih	*Lates calcarifer* Latidae	JF919828.1
7	Redspot emperor/ Landok calit merah	*Lethrinus lentjan* Lethrinidae	MF123937.1
8	Fork-tailed threadfin bream/Kerisi	*Nemipterus furcosus* Nemipteridae	KY371807.1
9	Cinnabar goatfish/ Biji nangka	*Parupeneus heptacanthus* Mullidae	KY371924.1
10	Parrot fish/ Bayan belang biru	*Scarus ghobban* Scaridae	JF494439.1
11	Monogrammed monocle bream/Pasir-pasir jalur jeap	*Scolopsis monogramma* Nemipterida	KP195008.1
12	Bigeye scad/ Lolong jalur	*Selar crumenophthalmus* Carangidae	KY372157.1
13	Longfin tuna/ Tuna sirip kuning	*Thunnus alalunga* Scombridae	KT074102.1

**Table 2:** Concentrations of non-essential metals in the fillets of different Malaysian fish species (mean mg/kg of fillet ± SD).

	Name	Be	Cd	Hg	Sb	Pb
1	*Caesio* *caerulaurea*	<0.2	<0.02	<0.01	<0.02	0.033 ± 0.007
2	*Caranx* *sexfasciatus*	<0.2	<0.02	<0.01	<0.02	0.024 ± 0.005
3	*Epinephelus* *aerolatus*	<0.2	<0.02	0.016 ± 0.003	<0.02	0.039 ± 0.008
5	*Johnius* *macrorhynus*	<0.2	<0.02	0.024 ± 0.005	<0.02	<0.02
6	*Lates* *calcarifer*	<0.2	<0.02	<0.01	<0.02	<0.02
7	*Lethrinus* *lentjan*	<0.2	<0.02	<0.01	<0.02	0.10 ± 0.02
8	*Nemipterus* *furcosus*	<0.2	<0.02	0.090 ± 0.018	<0.02	0.031 ± 0.006
9	*Parupeneus* *heptcanthus*	<0.2	<0.02	0.023 ± 0.005	<0.02	0.025 ± 0.005
10	*Scarus* *ghobban*	<0.2	<0.02	<0.01	<0.02	0.021 ± 0.004
11	*Scolopsis* *monogramma*	<0.2	<0.02	0.015 ± 0.003	<0.02	0.059 ± 0.012
12	*Selar* *crumneuphthalmus*	<0.2	<0.02	<0.01	<0.02	<0.02
13	*Thunnus* *alalunga*	<0.2	<0.02	<0.01	<0.02	0.033 ± 0.007
Malaysian MTL ^1^		1.0	1.0	1.0	1.0	1.0
European MTL ^2^		---	0.05	0.50	---	0.30

^1^ Maximum tolerable limit (MTL) according to Malaysian Food Act 1983 and Regulation 1985. ^2^ Maximum tolerable limit (MTL) according to EU Commission Regulation No. 1881/2006.

**Table 3:** Concentrations of essential metals in the fillets of different Malaysian fish species (mean mg/kg of fillet ± SD).

	Name	Li	Al	V	Cr	Mn	Co	Ni	Cu	Zn	As	Se
1	*Caesio* *caerulaurea*	<0.2	<2	0.10 ± 0.02	0.10 ± 0.02	0.38 ± 0.08	0.020 ± 0.004	<0.2	2.2 ± 0.4	3.6 ± 0.7	2.0 ± 0.4	0.58 ± 0.12
2	*Caranx* *sexfasciatus*	<0.2	<2	0.10 ± 0.02	0.11 ± 0.02	0.27 ± 0.05	<0.02	<0.2	2.1 ± 0.4	2.8 ± 0.6	0.6 ± 0.1	0.40 ± 0.08
3	*Epinephelus* *aerolatus*	<0.2	<2	<0.1	0.08 ± 0.02	0.27 ± 0.05	<0.02	<0.2	2.1 ± 0.4	2.6 ± 0.5	2.9 ± 0.6	0.54 ± 0.11
5	*Johnius* *macrorhynus*	<0.2	6.5 ± 1.2	<0.1	0.11 ± 0.02	0.35 ± 0.07	0.020 ± 0.004	<0.2	1.4 ± 0.3	3.3 ± 0.6	1.0 ± 0.2	0.22 ± 0.04
6	*Lates* *calcarifer*	<0.2	<2	<0.1	0.07 ± 0.01	0.21 ± 0.04	<0.02	<0.2	0.9 ± 0.2	2.7 ± 0.5	0.3 ± 0.1	<0.2
7	*Lethrinus* *lentjan*	<0.2	<2	<0.1	0.03 ± 0.01	<0.2	<0.02	<0.2	0.7 ± 0.1	1.6 ± 0.3	1.2 ± 0.2	0.23 ± 0.04
8	*Nemipterus* *furcosus*	<0.2	2.1 ± 0.4	<0.1	0.10 ± 0.02	0.41 ± 0.08	<0.02	<0.2	2.7 ± 0.5	3.5 ± 0.7	4.3 ± 0.8	0.79 ± 0.16
9	*Parupeneus* *heptcanthus*	<0.2	2.9 ± 0.6	<0.1	0.10 ± 0.02	0.22 ± 0.04	<0.02	<0.2	1.7 ± 0.3	3.6 ± 0.7	2.4 ± 0.5	0.36 ± 0.07
10	*Scarus* *ghobban*	<0.2	<2	<0.1	0.04 ± 0.01	<0.2	0.020 ± 0.004	<0.2	0.7 ± 0.1	1.9 ± 0.4	1.9 ± 0.4	0.62 ± 0.12
11	*Scolopsis* *monogramma*	<0.2	4.1 ± 0.8	0.10 ± 0.02	0.11 ± 0.02	0.68 ± 0.14	0.020 ± 0.004	0.28 ± 0.06	2.1 ± 0.4	5.7 ± 1.1	1.7 ± 0.3	0.51 ± 0.10
12	*Selar crumneuphthalmus*	<0.2	<2	<0.1	0.09 ± 0.02	0.20 ± 0.04	<0.02	<0.2	1.7 ± 0.3	3.0 ± 0.6	0.8 ± 0.2	0.31 ± 0.06
13	*Thunnus* *alalunga*	<0.2	<2	<0.1	0.09 ± 0.02	0.26 ± 0.05	<0.02	<0.2	2.6 ± 0.5	5.8 ± 1.2	0.4 ± 0.1	0.95 ± 0.19
	Malaysian MTL ^1^	---	---	---	---	---	---	---	---	---	1 ^#^	---
	European MTL ^2^	---	---	---	---	---	---	---	---	---	---	---

^1^ Maximum tolerable limit (MTL) according to Malaysian Food Act 1983 and Regulation 1985: metals not allowed. ^2^ Maximum tolerable limit (MTL) according to EU Commission Regulation No. 1881/2006: metals not allowed. ^#^ This value indicates inorganic arsenic.

**Table 4:** Total fat, total FA, SFA, MUFA, and PUFA concentration (mean mg/100 g of tissue ± SE) in fillets of Malaysian edible fishes.

		a	b	c	d	e	f	g	h
	Species	Total Lipids (g/100 g)	Total FA ^a^ (mg/100 g)	SFA ^b^ (mg/100 g)	MUFA ^c^ (mg/100 g)	PUFA ^d^: ω-3 ^e^ + ω-6 ^f^ FA (mg/100 g)	ω-3 ^e^ FA (mg/100 g)	ω-3/ω-6 PUFA Ratio	PUFA/SFA Ratio
1	*Caesio* *caerulaurea*	0.67 ± 0.04	104.7 ± 7.6	61.1 ± 2.3	18.2 ± 1.8	25.4 ± 3.5	19.1 ± 2.5	3.1 ± 0.9	0.41 ± 0.10
2	*Caranx* *sexfasciatus*	0.90 ± 0.05	151.6 ± 7.8	80.0 ± 2.2	43.1 ± 2.5	28.4 ± 3.1	23.0 ± 2.5	4.2 ± 0.9	0.36 ± 0.06
3	*Epinephelus* *areolatus*	0.84 ± 0.04	29.2 ± 3.8	14.2 ± 1.7	4.5 ± 0.1	10.5 ± 2.0	7.2 ± 1.6	2.2 ± 0.8	0.74 ± 0.16
4	*Himantura* *walga*	1.12 ± 0.06	636.5 ± 47.7	360.2 ± 23.8	105.6 ± 9.3	170.7 ± 14.6	127.8 ± 4.4	3.0 ± 0.8	0.47 ± 0.08
5	*Johnius* *macrorhynus*	1.16 ± 0.05	474.6 ± 25.7	252.2 ± 13.4	134.5 ± 2.0	88.0 ± 10.2	58.9 ± 6.1	2.0 ± 0.5	0.35 ± 0.05
6	*Latscalcarifer*	1.56 ± 0.06	672.8 ± 46.3	221.8 ± 9.5	241.4 ± 23.6	209.6 ± 13.2	112.0 ± 4.3	1.2 ± 0.2	0.95 ± 0.15
7	*Lethrinus* *lentjan*	1.14 ± 0.05	384.4 ± 20.7	197.5 ± 11.5	97.3 ± 2,7	89.6 ± 6.4	60.6 ± 4.0	2.1 ± 0.3	0.45 ± 0.05
8	*Nemipterus* *furcosus*	1.06 ± 0.05	64.0 ± 1.9	30.1 ± 0.6	7.9 ± 0.4	26.0 ± 0.6	22.9 ± 0.5	7.4 ± 0.4	0.87 ± 0.07
9	*Parupeneus* *heptacanthus*	0.57 ± 0.03	125.8 ± 10.7	72.3 ± 4.9	16.0 ± 1.4	37.5 ± 4.4	23.9 ± 2.4	1.8 ± 0.44	0.52 ± 0.11
10	*Scaurus* *ghobban*	0.85 ± 0.04	239.7 ± 27.9	110.0 ± 4.6	56.3 ± 9.4	73.4 ± 13.9	44.1 ± 10.5	1.5 ± 0.5	0.67 ± 0.24
11	*Scolopsis* *monogramma*	0.64 ± 0.04	197.5 ± 10.7	63.9 ± 3.0	31.4 ± 1.2	102.22 ± 6.46	70.47 ± 5.29	2.2 ± 0.2	1.60 ± 0.16
12	*Selar* *crumenophthalmus*	1.84 ± 0.07	400.30 ± 15.82	226,.2 ± 5.2	78.0 ± 4.4	96.0 ± 6.2	76.3 ± 3.6	3.9 ± 0.7	0.42 ± 0.05
13	*Thunnus* *alalunga*	1.14 ± 0.05	233.80 ± 9.03	85.1 ± 2.1	40.2 ± 1.6	108.5 ± 5.4	95.4 ± 4.5	7.3 ± 0.8	1.28 ± 0.11

^a^: Total fatty acids recovered from fillets. ^b^: Total SFAs in fillets, including C14:0 (myristic acid), C15:0 (pentadecylic acid), C16:0 (palmitic acid), C17:0 (margaric acid), C18:0 (Stearic acid), C19:0 (nonadecylic acid), C20:0 (arachidic acid), and C22:0 (behenic acid). ^c^: Total of all MUFAs in fillets, including C16:1ω-7 (palmitoleic acid), C17:1; C18:1 (elaidic acid (C18:1 ω-7) or vaccenic acid (C18:1ω-7), and C18:1ω-9 (oleic acid). ^d^: Total PUFAs in fillets; the most abundant were evaluated, and they corresponded to ω-3 and ω-6 fatty acids. ^e^: Total ω-3 fatty acids in fillets, including C20:5ω-3 (EPA) and C22:6ω-3 (DHA). ^f^: Total ω-6 fatty acids in fillets, including 18:2ω-6 (linoleic acid) and 20:4ω-6 (arachidonic acid) and traces of α-linolenic acid (ALA, C18:3 ω-3).

## Supplementary Materials

The following are available online at https://www.mdpi.com/article/10.3390/foods10102456/s1, Table S1. Fish species identified in Kuala Terengganu, Malaysia. Figure S1. Sites (1–5) of fillet collection. Fillet aliquots of this study were obtained from site 3.

## References

[1] Sawe B.E. Native Fish of Malaysia. https://www.worldatlas.com/articles/native-fish-of-malaysia.html.

[2] Malaysian Fisheries Society: MFS. http://www.mfs.org.my.

[3] Mazzeo M.F., Giulio B.D., Guerriero G., Ciarcia G., Malorni A., Russo G.L., Siciliano R.A. (2008). Fish Authentication by MALDI-TOF Mass Spectrometry. J. Agric. Food Chem..

[4] Di Finizio A., Guerriero G., Russo G.L., Ciarcia G. (2007). Identification of Gadoid Species (Pisces, Gadidae) by Sequencing and PCR–RFLP Analysis of Mitochondrial 12S and 16S RRNA Gene Fragments. Eur. Food Res. Technol..

[5] Guerriero G., Rabbito D., Alwany M.A., Madonna A., Temraz T.A., Sulaiman O.O., Bassem S.M., Trocchia S., Abdel-Gawad F.K., Ciarcia G. (2017). Fisheries and Biodiversity along Mediterranean Sea: Italian and Egyptian Coast Overview. Euro-Mediterr. J. Environ. Integr..

[6] Jacobsen C., García-Moreno P.J., Yesiltas B., Sørensen A.-D.M. (2021). Lipid Oxidation and Traditional Methods for Evaluation. Omega-3 Delivery Systems.

[7] Félix R., Valentão P., Andrade P.B., Félix C., Novais S.C., Lemos M.F.L. (2020). Evaluating the in vitro Potential of Natural Extracts to Protect Lipids from Oxidative Damage. Antioxidants.

[8] Slivinska L.G., Shcherbatyy A.R., Lukashchuk B.O., Gutyj B.V. (2020). The State of Antioxidant Protection System in Cows under the Influence of Heavy Metals. Regul. Mech. Biosyst..

[9] Leonard S.S., Harris G.K., Shi X. (2004). Metal-Induced Oxidative Stress and Signal Transduction. Free Radic. Biol. Med..

[10] Guerriero G., Trocchia S., Abdel Gawad F.K., Ciarcia G. (2014). Roles of reactive oxygen species in the spermatogenesis regulation. Front. Endocrinol..

[11] Rikans L.E., Hornbrook K.R. (1997). Lipid Peroxidation, Antioxidant Protection and Aging. Biochim. Biophys. Acta BBA Mol. Basis Dis..

[12] Viarengo A., Canesi L., Pertica M., Poli G., Moore M.N., Orunesu M. (1990). Heavy Metal Effects on Lipid Peroxidation in the Tissues of Mytilus galloprovincialis Lam. Comp. Biochem. Physiol. Part C Comp. Pharmacol..

[13] Velasco J., Dobarganes C., Márquez-Ruiz G., Skibsted L.H., Risbo J., Andersen M.L. (2010). Oxidative Rancidity in Foods and Food Quality. Chemical Deterioration and Physical Instability of Food and Beverages.

[14] Guerriero G., Bassem S.M., Khalil W.K.B., Temraz T.A., Ciarcia G., Abdel-Gawad F.K. (2018). Temperature changes and marine fish species (*Epinephelus coioides* and *Sparus aurata*): Role of oxidative stress biomarkers in toxicological food studies. Emir. J. Food Agric..

[15] Abdel-Gawad F.K., Khalil W.K.B., Bassem S.M., Kumar V., Parisi C., Inglese S., Temraz T.A., Nassar H.F., Guerriero G. (2020). The duckweed, *Lemna minor* modulates heavy metal-induced oxidative stress in the Nile tilapia *Oreochromis niloticus*. Water.

[16] Swanson D., Block R., Mousa S.A. (2012). Omega-3 Fatty Acids EPA and DHA: Health Benefits throughout Life. Adv. Nutr..

[17] Luchtman D.W., Song C. (2013). Cognitive Enhancement by Omega-3 Fatty Acids from Childhood to Old Age: Findings from Animal and Clinical Studies. Neuropharmacology.

[18] Lavie C.J., Milani R.V., Mehra M.R., Ventura H.O. (2009). Omega-3 Polyunsaturated Fatty Acids and Cardiovas-Cular Diseases. J. Am. Coll. Cardiol..

[19] Djoussé L., Akinkuolie A.O., Wu J.H.Y., Ding E.L., Gaziano J.M. (2012). Fish Consumption, Omega-3 Fatty Acids and Risk of Heart Failure: A Meta-Analysis. Clin. Nutr..

[20] Chattipakorn N., Settakorn J., Petsophonsakul P., Suwannahoi P., Mahakranukrauh P., Srichairatanakool S., Chattipakorn S.C. (2009). Cardiac Mortality Is Associated with Low Levels of Omega-3 and Omega-6 Fatty Acids in the Heart of Cadavers with a History of Coronary Heart Disease. Nutr. Res..

[21] Oehlenschläger J. (2012). Seafood: Nutritional Benefits and Risk Aspects. Int. J. Vitam. Nutr. Res..

[22] Łuczyńska J., Paszczyk B. (2019). Health Risk Assessment of Heavy Metals and Lipid Quality Indexes in Freshwater Fish from Lakes of Warmia and Mazury Region, Poland. Int. J. Environ. Res. Public Health.

[23] Salini M.J., Wade N.M., Araújo B.C., Turchini G.M., Glencross B.D. (2016). Eicosapentaenoic Acid, Arachidonic Acid and Eicosanoid Metabolism in Juvenile Barramundi Lates calcarifer. Lipids.

[24] Connor S.L., Gustafson J.R., Sexton G., Becker N., Artaud-Wild S., Connor W.E. (1992). The Diet Habit Survey: A New Method of Dietary Assessment That Relates to Plasma Cholesterol Changes. J. Am. Diet. Assoc..

[25] Pal J., Shukla B.N., Maurya A.K., Verma H.O., Pandey G. (2018). Amitha A Review on Role of Fish in Human Nutrition with Special Emphasis to Essential Fatty Acid. Int. J. Fish. Aquat. Stud..

[26] Metillo E.B., Aspiras-Eya A.A. (2014). Fatty Acids in Six Small Pelagic Fish Species and Their Crustacean Prey from the Mindanao Sea, Southern Philippines. Trop. Life Sci. Res..

[27] Van der Meeren T., Olsen R.E., Hamre K., Fyhn H.J. (2008). Biochemical Composition of Copepods for Evaluation of Feed Quality in Production of Juvenile Marine Fish. Aquaculture.

[28] Jackson G.D., Bustamante P., Cherel Y., Fulton E.A., Grist E.P.M., Jackson C.H., Nichols P.D., Pethybridge H., Phillips K., Ward R.D. (2007). Applying New Tools to Cephalopod Trophic Dynamics and Ecology: Perspectives from the Southern Ocean Cephalopod Workshop, February 2–3, 2006. Rev. Fish Biol. Fish..

[29] Boecklen W.J., Yarnes C.T., Cook B.A., James A.C. (2011). On the Use of Stable Isotopes in Trophic Ecology. Annu. Rev. Ecol. Evol. Syst..

[30] Traugott M., Kamenova S., Ruess L., Seeber J., Plantegenest M. (2013). Empirically Characterising Trophic Networks: What Emerging DNA-Based Methods, Stable Isotope and Fatty Acid Analyses Can Offer. Adv. Ecol. Res..

[31] Daly E.A., Benkwitt C.E., Brodeur R.D., Litz M.N.C., Copeman L.A. (2010). Fatty Acid Profiles of Juvenile Salmon Indicate Prey Selection Strategies in Coastal Marine Waters. Mar. Biol..

[32] Arai T., Amalina R., Bachok Z. (2015). Diverse Feeding Ecology and Habitat Use in Coral Reef Fishes in the Malay-Sian South China Sea, as Revealed by Liver Fatty Acid Composition. Oceanol. Hydrobiol. Stud..

[33] Dhurmeea Z., Pethybridge H., Appadoo C., Bodin N. (2018). Lipid and Fatty Acid Dynamics in Mature Female Al-Bacore Tuna (*Thunnus alalunga*) in the Western Indian Ocean. PLoS ONE.

[34] Cladis D.P., Kleiner A.C., Freiser H.H., Santerre C.R. (2014). Fatty Acid Profiles of Commercially Available Finfish Fillets in the United States. Lipids.

[35] FAO International Symposium on Fisheries Sustainability: Strengthening the Science-Policy Nexus, 18–21 November 2019. Rome. http://www.fao.org/3/ca6763en/CA6763EN.pdf.

[36] Azmi L.S. (2018). Physical and Chemical Characterization of Iron Ore from East Coast of Peninsular Malaysia. https://ethesis.usm.my/jspui/bitstream/123456789/12122/1/Physical%20and%20chemical%20characterization%20of%20iron%20ore%20from%20east%20coast%20of%20peninsular%20Malaysia_Latifah%20Suha%20Azmi_B1_2018_MJMS.pdf.

[37] FAO. http://www.fao.org/about/en/.

[38] WoRMS—World Register of Marine Species. https://www.marinespecies.org/about.php.

[39] FishBase: A Global Information System on Fishes. http://www.fishbase.org/home.htm.

[40] Inkscape. https://inkscape.org/it/.

[41] Ward R.D. (2009). DNA Barcode Divergence among Species and Genera of Birds and Fishes. Mol. Ecol. Resour..

[42] Madonna A., Alwany M.A., Rabbito R., Trocchia S., Labar S., Abdel-Gawad F.K., D’Angelo R., Gallo A., Guerriero G., Ciarcia G. (2015). Caves Biodiversity in the Marine Area of Riviera d’Ulisse Regional Park, Italy: Grotta Del Maresciallo Overview. J. Biodivers. Endanger. Species.

[43] Gentilucci M., Parisi C., Coppola M.R., Majdoubi F.-Z., Madonna M., Guerriero G. (2021). Influence of Mediterranean Sea Temperature Increase on Gaeta Gulf (Tyrrhenian Sea) Biodiversity. Proc. Zool. Soc..

[44] http://www.ncbi.nlm.nih.gov.

[45] Mount D.W. (2007). Using a FASTA Sequence Database Similarity Search. Cold Spring Harb. Protoc..

[46] Lettieri G., Notariale R., Carusone N., Giarra A., Trifuoggi M., Manna C., Piscopo M. (2021). New Insights into Alterations in PL Proteins Affecting Their Binding to DNA after Exposure of *Mytilus galloprovincialis* to Mercury—A Possible Risk to Sperm Chromatin Structure?. Int. J. Mol. Sci..

[47] Bligh E.G., Dyer W.J. (1959). A Rapid Method of Total Lipid Extraction and Purification. Can. J. Biochem. Physiol..

[48] Christie W.W. (1993). Preparation of Ester Derivatives of Fatty Acids for Chromatographic Analysis. Adv. Lipid Methodol..

[49] Tvrzická E. (2002). Analysis of Fatty Acids in Plasma Lipoproteins by Gas Chromatography–Flame Ionization Detec-Tion: Quantitative Aspects. Anal. Chim. Acta.

[50] Prashanth L., Kattapagari K.K., Chitturi R.T., Baddam V.R.R., Prasad L.K. (2015). A Review on Role of Essential Trace Elements in Health and Disease. J. NTR Univ. Health Sci..

[51] Fischer J. (2013). Fish Identification Tools for Biodiversity and Fisheries Assessments: Review and Guidance for Decision-Makers.

[52] Malaysia-Food-Act-1983. http://extwprlegs1.fao.org/docs/pdf/mal27309.pdf.

[53] Arai T., Amalina R., Bachok Z. (2015). Fatty Acid Composition Indicating Diverse Habitat Use in Coral Reef Fishes in the Malaysian South China Sea. Biol. Res..

[54] Arai T., Amalina R., Bachok Z. (2015). Variation in Fatty Acid Composition of the Bigeye Snapper Lutjanus lutjanus Collected in Coral Reef Habitats of the Malaysian South China Sea. J. Biol. Res..

[55] Gentilucci M., Moustafa A.A., Abdel-Gawad F.K., Mansour S.R., Coppola M.R., Caserta L., Inglese S., Pambianchi G., Guerriero G. (2021). Advances in Egyptian Mediterranean Coast Climate Change Monitoring. Water.

[56] Kamaruzzaman B.Y., Rina Z., John B.A., Jalal K.C.A. (2011). Heavy Metal Accumulation in Commercially Important Fishes of South West Malaysian Coast. Res. J. Environ. Sci..

[57] Ong M.C., Gan S.L. (2017). Assessment of Metallic Trace Elements in the Muscles and Fins of Four Landed Elasmobranchs from Kuala Terengganu Waters, Malaysia. Mar. Pollut. Bull..

[58] Ariano A., Musco N., Severino L., De Maio A., Tramice A., Tommonaro G., Damiano S., Genovese A., Olanrewaju O.S., Bovera F. (2021). Chemistry of Tropical Eucheumatoids: Potential for Food and Feed Applications. Biomolecules.

[59] Azaman F., Juahir H., Yunus K., Azid A., Kamarudin M.K.A., Toriman M.E., Mustafa A.D., Amran M.A., Che Hasnam C.N., Mohd Saudi A.S. (2015). Heavy Metal in Fish: Analysis and Human Health—A Review. J. Teknol..

[60] World Health Organization, Food and Agriculture Organization of the United Nations, International Atomic Energy Agency (1996). Trace Elements in Human Nutrition and Health.

[61] Agency for Toxic Substances and Disease Registry (2000). Toxicological Profile for Arsenic.

[62] Pizent A., Tariba B., Zivkovic T. (2012). Reproductive Toxicity of Metals in Men. Arh. Hig. Rada Toksikol..

[63] Yunus S.M., Hamzah Z., Ariffin N.A.N., Muslim M.B. (2014). Cadmium, Chromium, Copper, Lead, Ferrum and Zinc Levels in the Cockles (*Anadara granosa*) from Kuala Selangor, Malaysia. Malays. J. Anal. Sci..

[64] Strobel C., Jahreis G., Kuhnt K. (2012). Survey of n-3 and n-6 Polyunsaturated Fatty Acids in Fish and Fish Products. Lipids Health Dis..

[65] Vlahogianni T.H., Valavanidis A. (2007). Heavy-Metal Effects on Lipid Peroxidation and Antioxidant Defence Enzymes in Mussels *Mytilus galloprovincialis*. Chem. Ecol..

[66] Majdoubi F.-Z., Ouizgane A., Farid S., Mossetti L., Droussi M., Guerriero G., Hasnaoui M. (2021). Fry Survival Rate as a Predictive Marker of Optimal Production of Silver Carp (*Hypophthalmichthys molitrix*, Valenciennes 1844): A Biostatistical Study in Deroua Fish Farm, Morocco. Proc. Zool. Soc..

[67] Huynh M.D., Kitts D.D. (2009). Evaluating Nutritional Quality of Pacific Fish Species from Fatty Acid Signatures. Food Chem..

[68] Garrido S., Rosa R., Ben-Hamadou R., Cunha M.E., Chícharo M.A., Lingen C.D. (2008). Spatio-Temporal Variability in Fatty Acid Trophic Biomarkers in Stomach Contents and Muscle of Iberian Sardine (*Sardina pilchardus*) and Its Relationship with Spawning. Mar. Biol..

[69] Gibson R.A., Kneebone R., Kneebone G.M. (1984). Comparative Levels of Arachidonic Acid and Eicosapentaenoic Acid in Malaysian Fish. Comp. Biochem. Physiol. C Comp. Pharmacol. Toxicol..

[70] Rawdah T., Elfaer M. (1994). Fatty-Acid Composition of Three Commercially Important Fish of the Arabian Gulf. Food Chem..

[71] Manthey-Karl M., Lehmann I., Ostermeyer U., Schröder U. (2016). Natural Chemical Composition of Commercial Fish Species: Characterisation of Pangasius, Wild and Farmed Turbot and Barramundi. Foods.

[72] Rasmussen R.S., Morrissey M.T., Roblero J. (2008). Fatty Acid Composition of U.S. West Coast Albacore Tuna (*Thunnus alalunga*) and the Effects of Canning and Short-Term Storage. J. Aquat. Food Prod. Technol..

[73] Galindo A., Garrido D., Monroig Ó., Pérez J.A., Betancor M.B., Acosta N.G., Kabeya N., Marrero M.A., Bolaños A., Rodríguez C. (2021). Polyunsaturated Fatty Acid Metabolism in Three Fish Species with Different Trophic Level. Aquaculture.

[74] Rodrigues B.L., da Cruz Silva Canto A.C.V., da Costa M.P., da Silva F.A., Mársico E.T., Conte-Junior C.A. (2017). Fat-Ty Acid Profiles of Five Farmed Brazilian Freshwater Fish Species from Different Families. PLoS ONE.

[75] Fernandes C.E., da Silva Vasconcelos M.A., de Almeida Ribeiro M., Sarubbo L.A., Andrade S.A.C., de Melo Filho A.B. (2014). Nutritional and Lipid Profiles in Marine Fish Species from Brazil. Food Chem..

[76] Her Majesty’s Stationery Office (1994). Nutritional Aspects of Cardiovascular Disease.

[77] Crexi V.T., Monte M.L., de Souza Soares L.A., Pinto L.A.A. (2010). Production and Refinement of Oil from Carp (*Cyprinus carpio*) Viscera. Food Chem..

[78] European Food Safety Authority (EFSA) (2009). Labelling Reference Intake Values for n-3 and n-6 Polyunsaturated Fatty Acids: Labelling Reference Intake Values for n-3 and n-6 Polyunsaturated Fatty Acids. EFSA J..

