# Supplementary material for: Comparative Fatty Acid Profiling of Edible Fishes in Kuala Terengganu, Malaysia
Source: Foods. 2021 Oct 14;10(10):2456. doi: 10.3390/foods10102456 (PMC8535710; doi:10.3390/foods10102456)
Supplement: Supplementary file 1 [file foods-10-02456-s001.zip › foods-1379573-supplementary.pdf]

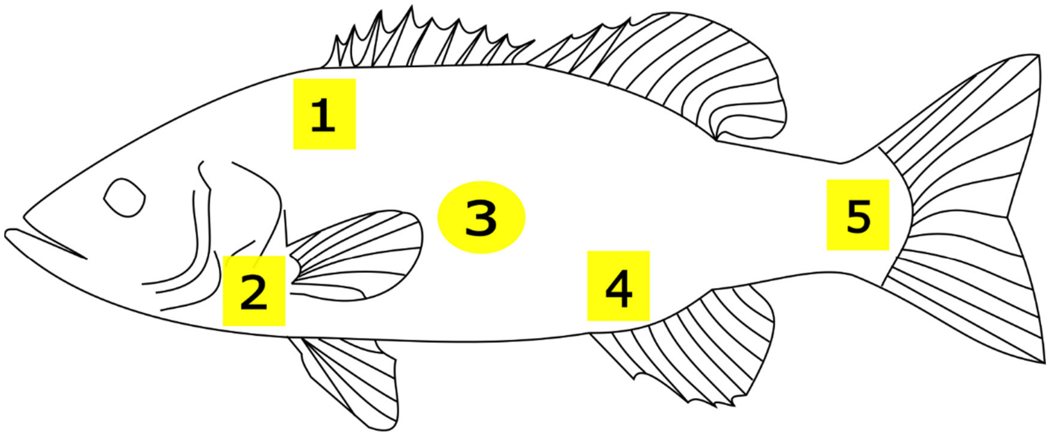

Figure S1. Sites (1-5) of fillet collection. Fillet aliquots of this study were obtained from site 3.

Table S1. Fish species identified in Kuala Terengganu, Malaysia. Photos by Gennaro Velotto. Schematic images created by Sara Inglese.

Fish and schematic image	Family characteristics	Genus characteristics	Species characteristics	Species identified
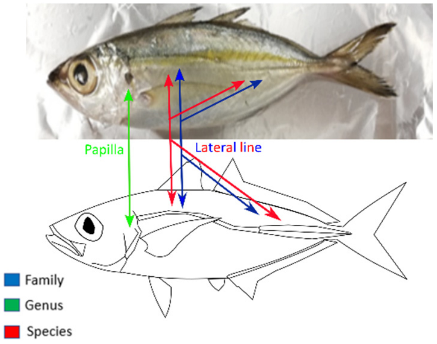	**Carangidae** (family): Lateral line elevated anteriorly and straight posteriorly, extending onto caudal fin	***Selar*** (genus): Shoulder girdle (cleithrum) margin with a furrow ventrally, a large papilla immediately above it and a smaller papilla near upper edge	***Selar crumenophthalmus*** (species): Curved part of lateral line with 48 to 56 scales; curved part of lateral line moderate, with chord of curved part contained 0.7 to 1.2 times in straight part; scutes smaller	*Selar crumenophthalmus*

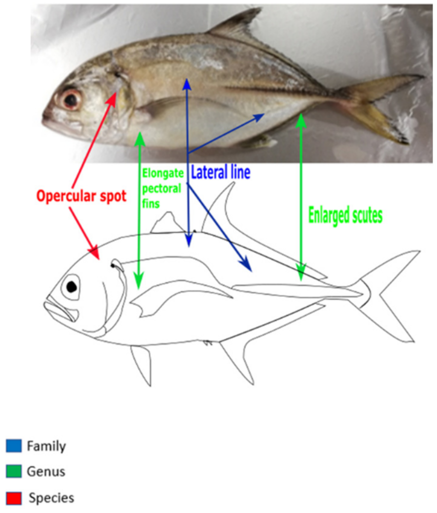	**Carangidae** (family): Lateral line elevated anteriorly and straight posteriorly, extending onto caudal fin	***Caranx*** (genus): Posterior straight part of lateral line with enlarged hardened scutes, pectoral fins long and falcate, longer than head.	***Caranx sexfasciatus*** (species): In adults, dorsal-fin lobe with white tip; dorsal profile of head moderately convex and black spot on upper margin of opercle, its size in adults no larger than twice the diameter of pupil, in specimens larger than 15 cm fork length, postorbital head length shorter, contained 6.4 to 8.2 times in fork length, and dorsal-fin lobe longer, contained 5 to 6.6 times in fork length; vertebrae 10+15	*Caranx sexfasciatus*
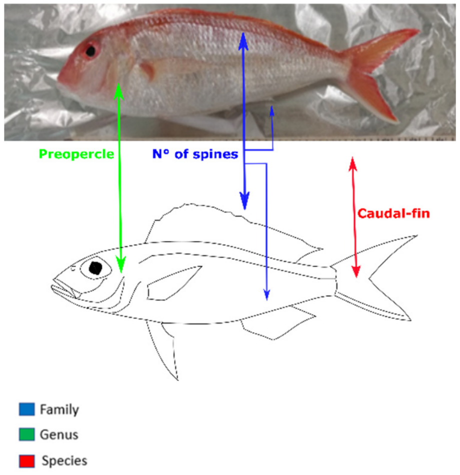	**Nemipteridae** (family): A single continuous dorsal fin, with X spines and 9 soft rays; anal fin with III spines and 7 soft rays	***Nemipterus*** (genus): Suborbital spine absent; 3 transverse scale rows on preopercle	***Nemipterus forcosus*** (species): Pelvic fins short, reaching to or short of anus; a line drawn upwards from posterior edge of the suborbital reaching the dorsal profile at about origin of dorsal fin; body pink, with darker saddles on back; caudal fin with lower margin white-edged; dorsal and anal fins without stripes.	*Nemipterus forcosus*

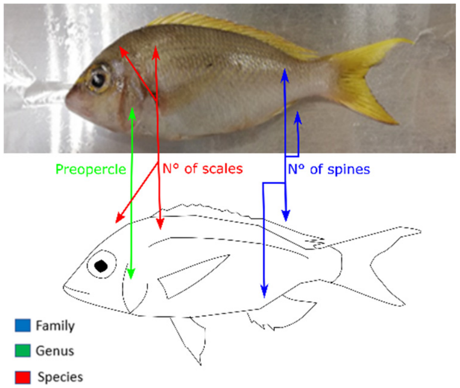	**Nemipteridae** (family): A single continuous dorsal fin, with X spines and 9 soft rays; anal fin with III spines and 7 soft rays	***Scolopsis*** (genus): Suborbital naked, with a large backwardly pointing spine and a series of smaller spines or serrations on its posterior margin; posterior margin of preopercle coarsely denticulate or serrate; canine teeth absent	***Scolopsis monogramma*** (species): Five or 6 transverse scale rows between lateral line and first dorsal-fin spine; scales on top of head not truncated anteriorly	*Scolopsis monogramma*
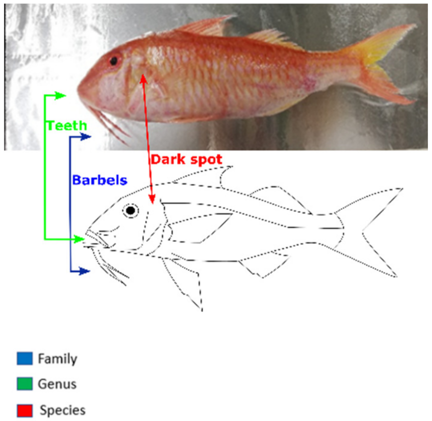	**Mullidae** (family): Two long unbranched barbels on chin Two well-separated dorsal fins, the first with 7 or 8 slender spines (first spine often very small), the second fin with 9 soft rays (first unbranched); anal fin with I spine and 6 or 7 soft rays; caudal fin deeply forked	***Parupeneus*** (genus): Teeth in jaws in 1 row, moderately large, bluntly conical, and well spaced; lateral-line scales 27 to 29; 2 or 3 scale rows between dorsal fins	***Parupeneus heptacanthus*** (species): Body less elongate, its depth 3 to 3.55 times in standard length; pectoral-fin rays 15 to 17; a dark reddish spot about the size of pupil usually present just below lateral line above outer third of pectoral fin; edges of scales dull orange-red on about upper three-fourths of body, white below, the scales of upper 4 rows with a pale bluish spot	*Parupeneus heptacanthus*

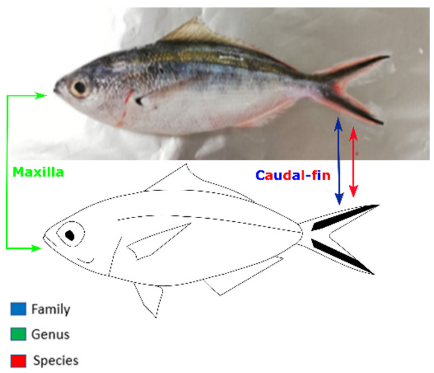	**Mullidae** (family): Dorsal fin with X to XV slender weak spines and 8 to 22 soft rays; anal fin with III spines and 9 to 13 soft rays; caudal fin distinctly forked, with pointed lobes	***Caesio*** (genus): A single postmaxillary process; posterior end of maxilla blunt, its greatest depth posterior to end of premaxilla	***Caesio caerulaurea*** (species): Lateral-line scales 57 to 65; scale rows on spinous portion of dorsal fin horizontal; caudal fin not yellow, each lobe with a median blackish streak	*Caesio caerulaurea*
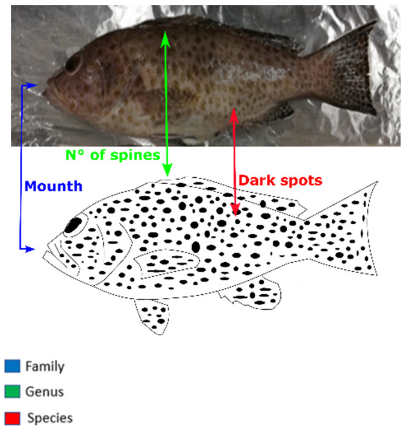	**Serranidae** (family): Opercle with 3 flat spines; margin of preopercle nearly always serrate or with 1 to 4 spines. Mouth large, terminal; maxilla exposed when mouth is closed; lower jaw usually projecting; bands of small, slender teeth in jaws; canines usually present at front of jaws and sometimes at side	***Epinephelus*** (genus) : Body depth 2.4 to 4.1 times in standard length, usually less than head length; dorsal fin with 11 spines and 12 to 19 soft rays, the base of soft-rayed part shorter than or equal to that of spinous part	***Epinephelus areolatus*** (species): Dorsal-fin rays 15 to 17; anal fin of adults rounded to slightly angular, the longest soft ray 2.0 to 2.6 times in head length; 14 to 16 gill rakers on lower limb of first gill arch; pyloric caeca 11 to 17; dark spots on body of adults about equal to pupil	*Epinephelus areolatus*

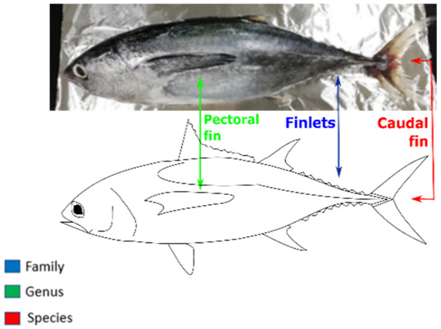	**Scombridae** (family): Finlets present behind dorsal and anal fins; caudal fin deeply forked	***Thunnus*** (genus): Body covered with very small scales behind corselet; no black spots on body; pectoral-fin rays 30 to 36	***Thunnus alalunga*** (species): Caudal fin with a narrow white posterior border; pectoral fins very long, reaching well past end of second dorsal-fin base; greatest body depth at or slightly before level of second dorsal fin	*Thunnus alalunga*
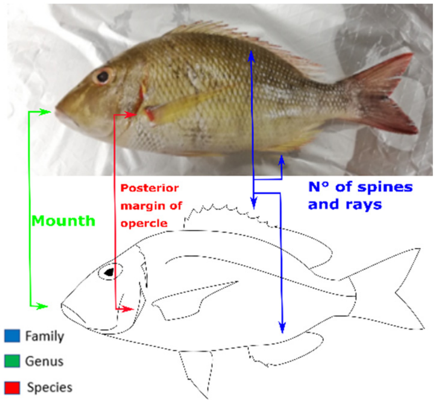	**Lethrinidae** (family): Dorsal fin continuous, with X spines and 9 or 10 soft rays; anal fin with III spines and 8 to 10 soft rays	***Lethrinus*** (genus): Cheek naked; 9 soft rays in dorsal fin; 8 soft rays in anal fin	***Lethrinus lentjan*** (species): In adults, lateral teeth in jaws with molars, molars with tubercles, or broadly rounded; body depth 2.5 to 2.9 times in standard length; posterior margin of opercle red and base of pectoral fins often red in life	*Lethrinus lentjan*

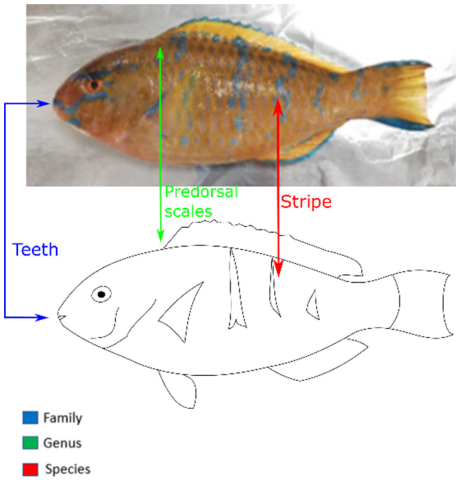	**Scaridae** (family): Teeth fused to form a pair of beak-like plates in each jaw, some species fused at base with individual teeth clearly visible, others with teeth visible at margins of tooth plates; large and heavy scales in regular rows on the head and body; pharyngeal dentition unique	***Scarus*** (genus): Jaws with overbite; median predorsal scales 6 or 7	***Scarus ghobban*** (species): Pink central stripe on dorsal and anal fins; blue markings around pectoral fin and underside often yellowish to pinkish.	*Scarus ghobban*
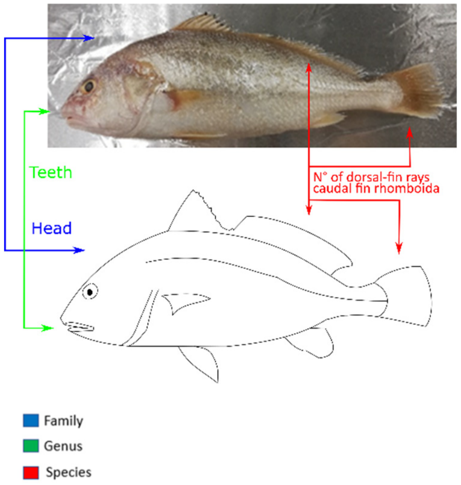	**Scianidae** (family): Moderately elongate, moderately compressed, small to large perciform fishes. Head and body completely scaly, except tip of snout; lateral-line scales extending to hind margin of caudal fin. Dorsal side of head (skull) cavernous with a series of bridge-like bony struts	***Johnius*** (genus): Lower jaw teeth uniform in size or inner row of lower jaw teeth molariform	***Johnius macrorhynus*** (species): Dorsal-fin rays 29 to 34; scales on body ctenoid; caudal fin rhomboida	*Johnius macrorhynus*

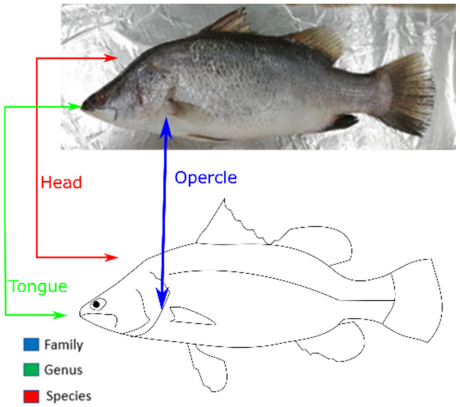	**Latidae** (family): Elongate and compressed body with a concave part at nape. Largemouth with jaws in the low side longer than the upper. Small teeth on jaws but also on the tongue sometimes, canine teeth present in some. Preopercle with serrated border or with two ridges, the opercle presents one spines only.	***Lates*** (genus): Upper jaw reaching to behind eye; nostrils close together; lower edge of preopercle serrated; tongue smooth; lower gillrakers 16 or 17	***Lates calcarifer*** (species): Head pointed, with concave dorsal profile becoming convex in front of dorsal fin	*Lates calcarifer*

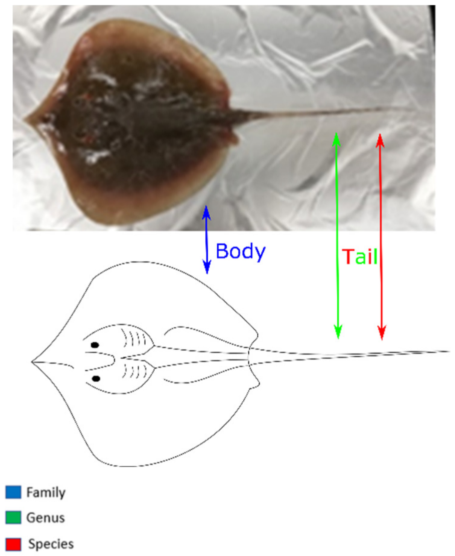	**Dasyatidae** (family): Body variably depressed with a well-formed oval, circular or rhombic disc that fully incorporates head; snout angular to obtuse and sometimes very elongate; nasal curtain well developed, skirt-shaped, rectangular or bilobed; five gill slits; oral papillae usually present on floor of mouth; tail moderately stout to slender-based and more or less elongated	***Brevitrygon*** *(genus):* Strongly depressed, oval to suboval disc with pectoral-fin apex broadly rounded; snout acutely angular and elongate (1.9–3.6 times combined orbit and spiracle length); eye small and protruding slightly; nasal curtain broadly skirt shaped; mouth medium-sized with 2–4 oral papillae; tail rather short and semi-rigid or filamentous distally (length 1–2.2 times DW), its base broad and strongly or weakly depressed; pelvic fins small to medium-sized, protruding slightly beyond disc	***Brevitrygon walga*** (species): Tail of male and juvenile filamentous, without pronounced lateral keels; tail of adult female bulbous beyond sting; thorns on tail very elongate, much longer than 1/2 eye diameter	*Brevitrygon walga*
